# Immediate and Delayed Mortality of Four Stored-Product Pests on Concrete Surfaces Treated with Chlorantraniliprole

**DOI:** 10.3390/insects12121088

**Published:** 2021-12-04

**Authors:** Nickolas G. Kavallieratos, Maria C. Boukouvala, Erifili P. Nika, Nikoleta Eleftheriadou, Dimitrios N. Avtzis

**Affiliations:** 1 Laboratory of Agricultural Zoology and Entomology, Department of Crop Science, Agricultural University of Athens, 75 Iera Odos Str., 11855 Athens, Attica, Greece; erifilinika@aua.gr; 2 Forest Research Institute, Hellenic Agricultural Organization Demeter, 57006 Vassilika, Thessalonıki, Greece; eleftheria6363@gmail.com (N.E.); dimitrios.avtzis@fri.gr (D.N.A.)

**Keywords:** anthranilic diamide, concrete surface, insect, mite, pests

## Abstract

**Simple Summary:**

We examined the mortality caused by the anthranilic diamide, chlorantraniliprole, at four different doses applied on concrete (0.01, 0.05, 0.1, and 0.5 mg a.i./cm^2^) in *Tribolium castaneum* (Herbst) (Coleoptera: Tenebrionidae) adults and larvae, *Rhyzopertha dominica* (F.) (Coleoptera: Bostrychidae) adults, *Sitophilus oryzae* (L.) (Coleoptera: Curculionidae) adults, and *Acarus siro* L. (Sarcoptiformes: Acaridae) adults and nymphs. Mortality data were recorded after 1, 2, 3, 4, and 5 days to determine the immediate mortality. Furthermore, after the 5-day mortality counts, still living individuals were conveyed for 7 days to untreated concrete surfaces to estimate the delayed mortality. The highest immediate mortality was recorded for the larvae of *T. castaneum*, reaching 96.7%, followed by the adults of *A. siro* (92.2%) after 5 days of exposure to 0.5 mg a.i./cm^2^. Complete (100.0%) delayed mortality was noticed for *T. castaneum* (adults and larvae), *S. oryzae*, and *A. siro* (both as adults) at 0.5 mg a.i./cm^2^. *Rhyzopertha dominica* adults and *A. siro* nymphs exhibited 98.6% and 96.3% delayed mortality at the same dose, respectively. Overall, our results demonstrate that chlorantraniliprole is effective against all the species tested, causing varying immediate and delayed mortality rates at the developmental stages tested.

**Abstract:**

Chlorantraniliprole is an effective pesticide against a plethora of pests, but its efficacy against stored-product pests is very poorly explored. In this study we treated concrete surfaces with four different doses of chlorantraniliprole (0.01, 0.05, 0.1, and 0.5 mg a.i./cm^2^) against the red flour beetle, *Tribolium castaneum* (Herbst) (Coleoptera: Tenebrionidae) adults and larvae, the lesser grain borer, *Rhyzopertha dominica* (F.) (Coleoptera: Bostrychidae) adults, the rice weevil, *Sitophilus oryzae* (L.) (Coleoptera: Curculionidae) adults, and the flour mite, *Acarus siro* L. (Sarcoptiformes: Acaridae) adults and nymphs, to examine the immediate mortalities after 1, 2, 3, 4, and 5 days of exposure. Additionally, the delayed mortality of the individuals that survived the 5-day exposure was also evaluated after a further 7 days on untreated concrete surfaces. We documented high mortality rates for all tested species and their developmental stages. After 5 days of exposure to 0.5 mg a.i./cm^2^, *T. castaneum* larvae and *A. siro* adults exhibited the highest immediate mortality levels, reaching 96.7% and 92.2%, respectively. Delayed mortality was also very high for all tested species and their developmental stages. Nymphs of *A. siro* displayed a 96.3% delayed mortality followed by the adults of *R. dominica* (98.6%) after exposure to 0.5 mg a.i./cm^2^. All other tested species and their developmental stages reached complete (100.0%) delayed mortality, where even 0.01 mg a.i./cm^2^ caused ≥86.6% delayed mortality in all species and their developmental stages. Taking into consideration the effectiveness of chlorantraniliprole on this wide range of noxious arthropods, coupled with its low toxicity towards beneficial arthropods and mammals, this pesticide could provide an effective management tool for stored-product pests in storage facilities.

## 1. Introduction

The red flour beetle, *Tribolium castaneum* (Herbst) (Coleoptera: Tenebrionidae) is a serious insect pest of high economic importance, infesting several stored cereals and foodstuffs worldwide [1]. It is a secondary pest commonly found in mills, warehouses, grocery stores, bakeries, and pet stores [2,3,4,5]. The lesser grain borer, *Rhyzopertha dominica* (F.) (Coleoptera: Bostrychidae) seems to have been originally feeding on dried fruits and forest trees [6], and it is currently identified as one of the most destructive insect pests of stored products globally [2,6,7]. As a primary pest, its larvae and adults can attack the sound kernels in storage facilities, while it can occasionally infect ripe grains in the field [8]. The rice weevil, *Sitophilus oryzae* (L.) (Coleoptera: Curculionidae) is a major pest of stored products worldwide, capable of infesting a wide range of commodities such as wheat, maize, barley, sorghum, rye, oats, rice, millet, cottonseed, dallisgrass seed, vetch seed, beans, nuts, flour, pasta, and cassava [2,6,8]. As this species is an internal feeder and completes its entire development inside kernels [6], its larvae remain protected from contact insecticides applied on the external part of the grain [9]. The flour mite, *Acarus siro* L. (Sarcoptiformes: Acaridae) is the most significant mite, infesting mainly cereal products like flour and other commodities like cheese, hay, medicinal herbs, spices, baby food, and fishmeal [8,10,11,12]. It can be found worldwide in farms, warehouses, mills, and empty grain bins, tainting the products with a musty smell [8,12]. Based on the biological traits presented before, the successful management of these species is restrained by specific difficulties. For instance, several insecticides are not effective against *R. dominica* that has developed resistance to specific formulations (including phosphine) [7,13,14,15,16,17,18,19]. Similarly, *S. oryzae* has become resistant to many insecticides in different parts of the world [19,20], indicating that the management of this species in storage facilities requires special care. It is well documented that *T. castaneum* presents resistance to a wide spectrum of insecticides around the globe [20,21,22,23,24]. Finally, *A. siro* is resistant to numerous pesticides. For instance, etrimfos, pirimiphos-methyl, fenitrothion, and chlorpyrifos-methyl were not totally (100%) efficient against *A. siro* individuals throughout a 36-week-long trial [25], while, similarly, beta-cyfluthrin, deltamethrin, chlorpyrifos, and a mix of *s*-bioallethrin and deltamethrin did not result in killing *A. siro* individuals after 21 days of exposure [26]. *Acarus siro* can survive the exposure to diatomaceous earth (DE), such as 3 g/kg Dryacide, from a sample taken from the surface of the bins, for up to 40 weeks [27]. As it becomes evident, the need to examine and find new alternative insecticidal active ingredients against these stored-product pests is imperative.

Chlorantraniliprole is a novel insecticide that belongs to the chemical group of anthranilic diamides [28,29]. It has a unique mode of action, activating the ryanodine receptor in insects’ muscles and releasing the cellular calcium that causes the termination of feeding, increased lethargy, the paralysis of muscles, eventually leading to death [29,30,31,32]. In addition, chlorantraniliprole presents low mammalian toxicity and can kill a wide range of insect pests without harming beneficial arthropods [30,31,33,34]. For instance, it is very effective against species of agricultural importance belonging to different orders, e.g., Coleoptera, Lepidoptera, Hemiptera, Diptera, Isoptera, and Thysanoptera [35,36,37,38,39,40,41]. Furthermore, recent studies have verified the insecticidal activity of chlorantraniliprole applied on different commodities (i.e., barley, maize, oats, peeled rice, whole rice, and wheat) against the Mediterranean flour moth, *Ephestia kuehniella* Zeller (Lepidoptera: Pyralidae), the psocid, *Liposcelis bostrychophila* Badonnel (Psocoptera: Liposcelididae), *R. dominica*, *S. oryzae*, and the confused flour beetle, *Tribolium confusum* Jacquelin du Val (Coleoptera: Tenebrionidae) [42], or on maize against the larger grain borer, *Prostephanus truncatus* (Horn) (Coleoptera: Bostrychidae) [43]. However, there is limited knowledge about the efficacy of chlorantraniliprole applied on surfaces against stored-product pests. For example, this compound was examined as a surface treatment on concrete against the different life stages (i.e., eggs, young and old larvae, pupae and adults) of *T. confusum* [44]. Therefore, the objective of the present study is to simultaneously evaluate the immediate and delayed mortality of chlorantraniliprole applied on concrete surfaces against four important stored-product pests, i.e., *T. castaneum* adults and larvae, *S. oryzae* adults, *R. dominica* adults, and *A. siro* adults and nymphs.

## 2. Materials and Methods

### 2.1. Insect and Mite Species

The insect species used in the bioassays were obtained from colonies maintained under laboratory conditions since 2003. *Tribolium castaneum* was cultured on wheat flour containing 5% brewer’s yeast at 25 °C and at 65% relative humidity in continuous darkness [45]. Adults of this species were <2 weeks old and larvae were 3rd–4th instar [46]. *Rhyzopertha dominica* and *S. oryzae* were reared on whole wheat at 25 °C at 65% relative humidity, and <2-week-old adults of these species were examined [47]. The initial population of *A. siro* was collected from wheat in Greek storage facilities in 2004, and since then it has been reared under laboratory conditions. The *A. siro* rearing medium consisted of a mixture of oat flakes, wheat germ, and the extract of dried yeast at a 10:10:1 *w*/*w* ratio. The colonies were kept at 25 °C and at 80% relative humidity. For the tests, the nymphs and adults of *A. siro* were selected according to their external morphology, i.e., the bodies of adults are larger and bear longer hairs than the bodies of nymphs [48]. All the above species were reared in continuous darkness.

### 2.2. Insecticidal Formulation

The formulation of chlorantraniliprole, Coragen^®^ SC (suspension concentrate) with a 200 g/L active ingredient a.i., which was provided by Dupont (Halandri, Greece), was used in the experiments.

### 2.3. Bioassays

Chlorantraniliprole was examined at four doses: 0.01, 0.05, 0.1, and 0.5 mg a.i./cm^2^. The experiments were carried out in a completely randomized block design, with three replicates and three subreplicates. Petri dishes that were 8 cm in diameter and 1.5 cm high, with a surface area of 50.27 cm^2^ each, were used. The concrete surface was made one day before the beginning of the tests by filling the bottoms of the Petri dishes with the CEM I 52.5 N material (Durostick, Aspropyrgos, Greece). To prevent the escape of the exposed individuals, the upper internal walls of all dishes were covered with polytetrafluoroethylene (60 wt% dispersion in water) (Sigma-Aldrich Chemie GmbH, Taufkirchen, Germany).

Spraying was conducted with the use of an AG-4 airbrush (Mecafer S.A., Valence, France), where 1 mL of an aqueous solution that contained the appropriate volume of the formulation corresponding to each dose was applied on concrete surface as a fine mist. The airbrush was cleaned with acetone after the spraying of each dose. A quantity of 0.5 g clean and pesticide-free whole wheat kernels without infestation was placed on each concrete surface after spraying, as food, for *R. dominica*, *S. oryzae*, and *A. siro*. For *T. castaneum*, the same quantity of soft white wheat flour (a variety mixture made from the endosperm only) was used in the experiments. An additional series of dishes were prepared and sprayed with distilled water (1 mL per dish) with a different AG-4 airbrush, as described above, to serve as controls. Subsequently, 10 individuals were transferred into each dish, and all dishes were put inside incubators set at 25 °C and at 65% relative humidity in continuous darkness. Mortality was determined under an Olympus stereomicroscope (Olympus SZX9, Bacacos S.A., Athens, Greece) after 1, 2, 3, 4, and 5 days of exposure in the treated dishes. After the 5th day of exposure, surviving individuals of each treated or untreated dish were transferred to new unsprayed concrete surfaces that also contained the same quantity of food for each species. The dishes were transferred again into the incubators with the same conditions for an additional period of 7 days. After this interval, the new dishes were opened and the number of the dead individuals per dish was counted, as described above.

### 2.4. Data Analysis

For all tested species, the immediate or delayed mortality of the controls was low (<5%), so no correction was considered necessary. The repeated measures model was used to separately analyze data for the immediate mortality of each tested species and developmental stage [49]. The repeated factor was the exposure interval, while mortality was the response variable. The dose was the main effect. Data for delayed mortality were submitted to a two-way ANOVA, with the species/developmental stage and dose as the main effects. The associated interaction of the main effects was also considered in the analysis. Prior to the analysis, the transformation of data to a log (x + 1) scale was carried out in order to normalize the variance [50,51]. Means were separated by the Tukey–Kramer honest significant difference (HSD) test at the 0.05 significance level [52]. All analyses were conducted using the JMP 14 software [53].

## 3. Results

### 3.1. Immediate Mortality

Between and within the exposure intervals, all main effects were significant for all tested species and life stages, except for *R. dominica* adults (Table 1).

The mortality of *T. castaneum* adults was very low after 3 days of exposure in all tested doses and did not exceed 35.6% at 0.5 mg a.i./cm^2^ (Table 2). Mortality increased further, reaching 56.7% at 0.5 mg a.i./cm^2^, while at the other doses it ranged between 30.0% and 44.4% after 4 days of exposure. One day later, chlorantraniliprole killed 81.1% of the exposed adults at the highest dose (0.5 mg a.i./cm^2^) while it caused moderate mortality at the lower doses (i.e., 0.01, 0.05, and 0.1 mg a.i./cm^2^), ranging from 42.2% to 61.1%.

After 2 days of exposure, the mortality of T. castaneum larvae remained at low levels, reaching 33.3% mortality (Table 3). One day later, mortality increased further, ranging between 21.1% and 37.8% at 0.01, 0.05, and 0.1 mg a.i./cm^2^, and 60.0% at 0.5 mg a.i./cm^2^. After 4 days of exposure, mortality reached 77.8% at 0.5 mg a.i./cm^2^, while at 0.05 and 0.1 mg a.i./cm^2^, it was moderate (48.9% and 58.9%, respectively). After 5 days of exposure, chlorantraniliprole killed almost all larvae (96.7%) at 0.5 mg a.i./cm^2^.

Regarding the immediate mortality of *S. oryzae*, after 3 days of exposure, it was very low at all tested doses and did not exceed 23.3% at 0.5 mg a.i./cm^2^ (Table 4). The overall mortality did not exceed 45.6% after 4 days of exposure. At 5 days post-exposure, 80.0% of the exposed adults died on concrete treated with 0.5 mg a.i./cm^2^, while mortality rates at all the other doses ranged between 45.6% and 71.1%.

After 4 days of exposure, the percentages of dead *R. dominica* adults was low for all tested doses, reaching 32.2% at 0.5 mg a.i./cm^2^ (Table 5). One day later, mortality increased without significant differences among doses, ranging from 34.4% to 50.0%.

The mortality levels of *A. siro* adults was very low at doses ≤0.1 mg a.i./cm^2^, reaching 38.9%, while at 0.5 mg a.i./cm^2^, moderate mortality was noted (54.4%) 3 days post-exposure (Table 6). One day later, mortality at 0.01 mg a.i./cm^2^ remained at low levels (38.9%), but at doses ≥0.1 mg a.i./cm^2^, it ranged between 50.0% and 72.2%. After 5 days of exposure, chlorantraniliprole killed 92.2% of the exposed adults at the highest dose, while at 0.01 mg a.i./cm^2^, moderate mortality was recorded (65.6%).

Regarding *A. siro* nymphs, after 3 days of exposure, mortality was low at all tested doses, reaching 46.7% at 0.5 mg a.i./cm^2^ (Table 7). Nymphal mortality ranged between 37.8% and 61.1% at 4 days post-exposure. Moderate mortality was recorded at doses ≥0.05 mg a.i./cm^2^, reaching 73.3% at 0.5 mg a.i./cm^2^ after 5 days of exposure.

### 3.2. Delayed Mortality of the Tested Species

The main effect dose was significant for all species (Table 8). Delayed mortality was high for *T. castaneum* adults, given that >91.0% of the adults died on concrete treated with 0.01, 0.05, or 0.1 mg a.i./cm^2^, while 100.0% was recorded at 0.5 mg a.i./cm^2^ (Table 9). The same trend was noted for larval delayed mortality of this species. Complete mortality was noticed at 0.5 mg a.i./cm^2^. Moreover, 100.0% delayed mortality was noted for *S. oryzae* adults at 0.1 and 0.5 mg a.i./cm^2^. *Rhyzopertha dominica* adults reached 96.7% and 98.6% delayed mortality at 0.1 and 0.5 mg a.i./cm^2^, respectively. Concerning *A. siro*, adults were more susceptible than nymphs. All adults died at 0.1 and 0.5 mg a.i./cm^2^. However, 94.4% and 96.3% of the exposed nymphs died on concrete treated with 0.1 and 0.5 mg a.i./cm^2^, respectively.

## 4. Discussion

Our results indicate that chlorantraniliprole is a promising pesticide for the effective control of the adults and larvae of *T. castaneum*, the adults of *S. oryzae* and *R. dominica*, and the adults and nymphs of *A. siro*, when applied on concrete surfaces. In the present study, chlorantraniliprole killed 81.1% of *T. castaneum* adults, 96.7% of *T. castaneum* larvae, 80.0% of *S. oryzae* adults, 50.0% of *R. dominica*, 92.2% of *A. siro* adults, and 73.3% of *A. siro* nymphs after 5 days of exposure to 0.5 a.i./cm^2^. In a former study, Kavallieratos et al. [42] investigated the efficacy of two chlorantraniliprole formulations (WG and SC) as grain protectants against *L. bostrychophila*, *R. dominica, S. oryzae* adults, *E. kuehniella* larvae, and *T. confusum* adults and larvae. The 10 mg a.i./kg of grain of the WG formulation killed 95.6% of *S. oryzae* adults after 7 days of exposure on treated whole rice, while at the same dose, the SC formulation caused the mortality of 99.4% exposed individuals. Both chlorantraniliprole formulations killed 100.0% of *S. oryzae* adults after 14 days of exposure to the 10 mg a.i./kg of grain. Concerning *R. dominica*, the WG formulation at 10 mg a.i./kg of grain killed 88.3% of the adults after 7 days of exposure and 100.0% after 14 days of exposure on whole rice and wheat, respectively. The SC chlorantraniliprole was also effective at causing 83.3% and 96.7% mortality rates 7 and 14 days post-exposure, respectively, to treated whole rice. Furthermore, both chlorantraniliprole formulations effectively controlled *E. kuehniella* larvae, *L. bostrychophila* adults, and *T. confusum* adults and larvae on several commodities (e.g., hard wheat, barley, maize, peeled rice, whole rice, and oats). Similarly, Saglam et al. [44] documented that 0.1 mg a.i./cm^2^ sprayed on concrete killed 66%, 100.0%, and 64% of *T. confusum* adults, young larvae, and old larvae, respectively. It also delayed the adult emergence on the first 5 days of the experiment. Chlorantraniliprole (WG and SC) is also effective as maize protectant against *P. truncatus*. In a recent study, Boukouvala and Kavallieratos [43] found that 10 mg a.i. WG/kg of grain and 10 mg a.i. SC/kg of grain killed 98.9% and 96.1% of the exposed individuals after 14 days of exposure, respectively, at 30 °C. Although chlorantraniliprole is not yet authorized for the management of stored-product pests, the aforementioned findings are valuable inputs for its potential use. These research efforts are of particular importance as the number of registered plant protection products in stored-product protection is considerably reduced [54]. Therefore, chlorantraniliprole can be clasified as an a.i. that could potentially recieve registration to be used in storage facilities as a grain protectant and/or as a structural treatment.

Surface treatments constitute an effective approach for the management of stored-product pests. To date, a plethora of pesticides have been applied onto several types of surfaces under different scenarios. For instance, when Kavallieratos et al. [55] treated polypropylene storage bags with alpha-cypermethrin, the immediate mortality of *R. dominica* and *S. oryzae* adults was 37.8% and 40.0% at 5 days post-exposure, respectively. Adults of *T. castaneum* that were exposed to plywood, tiles, and concrete treated with chlorfenapyr exhibited different levels of survival (40.0%, 25.5%, and 2.5%, respectively), concluding that the type of the treated surface affects the efficacy of the tested formulation [56]. Similarly, Vassilakos et al. [57] evaluated ceramic tiles, plywood, concrete, and galvanized steel treated with spinetoram against *T. confusum*, *S. oryzae*, *S. granarius*, *R. dominica*, the rusty grain beetle, *Cryptolestes ferrugineus* (Stephens) (Coleoptera: Laemophloeidae), and *Oryzaephilus surinamensis* (L.) (Coleoptera: Silvanidae) adults. The different treated surfaces led to different mortality rates among all the exposed pests. For example, 0.05 mg spinetoram/cm^2^ killed 77.4%, 68.1%, 89.0%, and 59.2% of *T. confusum* adults on ceramic tiles, plywood, concrete, and galvanized steel, respectively, at 7 days post-exposure. Furthermore, the mortality of *E. kuehniella* larvae caused by thiamethoxam, alpha-cypermethrin, and deltamethrin treated on woven polypropylene (0.10 mg a.i./cm^2^) ranged between 40.0% and 82.2% [58]. Our findings document that chlorantraniliprole caused even higher mortality rates than the aforementioned formulations in some of the stored-product pests, as in the cases of *T. castaneum* larvae and *A. siro* adults. The fact that chlorantraniliprole killed almost all young larvae of *T. castaneum* under the short exposure scenario followed in our study is of particular importance. This is because chlorantraniliprole can cause the rapid collapse of the larval population upon contact to treated concrete and can consequently prohibit the emergence of adults which, as flyers and walkers, easily colonize stored food commodities [8,59,60]. Whether chlorantraniliprole can perform similarly to the older larvae of *T. castaneum* merits further investigation. In an earlier study, Saglam et al. [44] showed that when the old larvae of *T. confusum* came into contact with concrete surfaces treated with chlorantraniliprole, they exhibited tolerance to this toxicant, taking into account that around 50% of larvae survived after 14 days of exposure. Our study provides new information about the management of *A. siro* on surface treatment, given that there is limited knowledge, mostly coming from previous decades. For example, the DEs SilicoSec and Diasecticide killed 28–98% and 64–88% of *A. siro* adults after 24 h of exposure to three slurry doses (2.5, 5, and 10 g/m^2^) [61]. Although we obtained low mortality rates after 24 h of exposure with *A. siro* adults, 92.2% finally died 5 days post-exposure, which is also considered a short exposure interval.

In our study, the delayed mortality was extremely high, ranging between 96.3% and 100.0% in all tested species and their developmental stages. This is an important finding as it provides a plausible scenario of the arthropods moving from treated to untreated areas that may lead to their colonization by the pests [55,62,63,64]. This highly toxic effect of delayed mortality is also evident in the cases of several other pesticides. For instance, alpha-cypermethrin caused moderate-to-high delayed mortality in *R. dominica* (from 37.8% to 46.7%) and *S. oryzae* adults (from 32.2% to 60.0%) [55]. Chlorfenapyr killed 56.7% of *S. oryzae* adults after being transferred onto untreated surfaces (polypropylene bags) [55]. As with immediate mortality, delayed mortality may differ depending on the surface that the pesticide is applied. For example, 0.1 mg spinetoram/cm^2^ killed 100.0% of *R. dominica* on concrete, but 97.0% on galvanised steel [57]. The presence of untreated food lowered the delayed mortality rates of *S. oryzae* adults from 100.0% to 98.8% and from 91.4% to 78.7% with the *T. confusum* adults [57]. In addition to the aforementioned parameters that affect the rates of delayed mortality, the species and the developmental stage of the exposed pest are equally important, something that was clearly demonstrated both in the current study and in previous investigations. For example, thiamethoxan killed 3.3% of *T. molitor* adults and 35.6% of *T. molitor* large larvae, while the same pesticide killed 63.3% of *T. granarium* adults and 1.1% of *T. granarium* old larvae [62].

## 5. Conclusions

In conclusion, our study provides new data towards the efficacy of chlorantraniliprole on concrete at four doses against important arthropod pests of stored products. On the basis of our findings, chlorantraniliprole can be a useful management tool since it caused high levels of both immediate and delayed mortality to the majority of the tested species and their developmental stages. However, further experimentation is required to assess the efficacy of chlorantraniliprole applied on other types of surfaces (e.g., storage bags) under different environmental conditions (temperature and relative humidity), doses, exposure intervals, and other stored-product pests in conjunction with their egg laying capacities.

## Figures and Tables

**Table 1:** MANOVA parameters for main effects and associated interaction for mortality of *Tribolim castaneum* adults and larvae, *Sitophilus oryzae* adults, *Rhyzopertha dominica* adults, and *Acarus siro* adults and nymphs between and within exposure intervals (error DF = 32).

Effect	DF	*T. castaneum*		*T. castaneum*		*S. oryzae*		*R. dominica*		*A. siro*		*A. siro*	
		Adults		Larvae		Adults		Adults		Adults		Nymphs	
Between exposure intervals													
Source		*F*	*p*	*F*	*p*	*F*	*p*	*F*	*p*	*F*	*p*	*F*	*p*
Intercept	1	4928	<0.01	1293.2	<0.01	2189.7	<0.01	447.9	<0.01	1587.6	<0.01	5554.6	<0.01
Dose	2	36.7	<0.01	9.0	0.01	8.8	0.01	3.6	0.02	10.7	<0.01	19.4	<0.01
Within exposure intervals													
Source		*F*	*p*	*F*	*p*	*F*	*p*	*F*	*p*	*F*	*p*	*F*	*p*
Exposure	4	6285.2	<0.01	112.6	<0.01	6638.0	<0.01	101.9	<0.01	83.2	<0.01	68.2	<0.01
Exposure × dose	8	9.1	<0.01	2.4	0.01	5.8	<0.01	0.7	0.75	3.1	0.01	2.7	0.01

**Table 2:** Mean immediate mortality (% ± SE) of *Tribolium* *castaneum* adults exposed on concrete treated with chlorantraniliprole at four doses (0.01, 0.05, 0.1, and 0.5 mg a.i./cm^2^), for 1, 2, 3, 4, and 5 days. Within each row, means followed by the same uppercase letter are not significantly different (in all cases DF = 4, 44, Tukey–Kramer HSD test at *p* = 0.05). Within each column, means that are followed by the same lower-case letter are not significantly different (in all cases DF = 3, 35, Tukey–Kramer HSD test at *p* = 0.05). Where no letters exist, no significant differences were recorded. Where dashes exist, no analysis was performed.

Exposure	1 Day	2 Days	3 Days	4 Days	5 Days	*F*	*p*
Dose (mg a.i./cm^2^)							
0.01	0.0 ± 0.0 D	0.0 ± 0.0 Db	12.2 ± 1.5 Cc	30.0 ± 1.7 Bc	42.2 ± 2.8 Ac	1045.9	<0.01
0.05	0.0 ± 0.0 D	0.0 ± 0.0 Db	18.9 ± 2.0 Cb	36.7 ± 3.3 Bbc	54.4 ± 2.9 Ab	790.0	<0.01
0.1	0.0 ± 0.0 C	2.2 ± 1.5 Cb	28.9 ± 3.1 Ba	44.4 ± 2.9 ABab	61.1 ± 3.1 Ab	132.0	<0.01
0.5	0.0 ± 0.0 D	11.1 ± 2.0 Ca	35.6 ± 2.9 Ba	56.7 ± 2.9 ABa	81.1 ± 1.1 Aa	160.0	<0.01
*F*	-	21.8	20.8	14.8	29.8		
*p*	-	<0.01	<0.01	<0.01	<0.01		

**Table 3:** Mean immediate mortality (% ± SE) of *Tribolium* *castaneum* larvae exposed on concrete treated with chlorantraniliprole at four doses (0.01, 0.05, 0.1, and 0.5 mg a.i./cm^2^) for 1, 2, 3, 4, and 5 days. Within each row, means followed by the same uppercase letter are not significantly different (in all cases DF = 4, 44, Tukey–Kramer HSD test at *p* = 0.05). Within each column, means that are followed by the same lower-case letter are not significantly different (in all cases DF = 3, 35, Tukey–Kramer HSD test at *p* = 0.05). Where no letters exist, no significant differences were recorded.

Exposure	1 Day	2 Days	3 Days	4 Days	5 Days	*F*	*p*
Dose (mg a.i./cm^2^)							
0.01	2.2 ± 1.5 Cb	7.8 ± 3.6 Cb	21.1 ± 3.5 Bc	38.9 ± 2.0 ABc	66.7 ± 2.4 Ac	30.3	<0.01
0.05	5.6 ± 1.8 Cab	13.3 ± 3.3 BCab	26.7 ± 2.9 ABbc	48.9 ± 3.1 Abc	77.8 ± 4.3 Abc	20.2	<0.01
0.1	8.9 ± 2.6 Cab	22.2 ± 4.0 BCa	37.8 ± 5.2 ABb	58.9 ± 5.1 Ab	84.4 ± 4.8 Aab	15.1	<0.01
0.5	14.4 ± 2.9 Ca	33.3 ± 4.1 Ba	60.0 ± 4.4 ABa	77.8 ± 4.0 Aa	96.7 ± 1.7 Aa	25.2	<0.01
*F*	4.3	5.8	12.2	20.0	12.3		
*p*	0.01	0.01	<0.01	<0.01	<0.01		

**Table 4:** Mean immediate mortality (% ± SE) of *Sitophilus oryzae* adults exposed on concrete treated with chlorantraniliprole at four doses (0.01, 0.05, 0.1, and 0.5 mg a.i./cm^2^), for 1, 2, 3, 4, and 5 days. Within each row, means followed by the same uppercase letter are not significantly different (in all cases DF = 4, 44, Tukey–Kramer HSD test at *p* = 0.05). Within each column, means that are followed by the same lower-case letter are not significantly different (in all cases DF = 3, 35, Tukey–Kramer HSD test at *p* = 0.05). Where no letters exist, no significant differences were recorded. Where dashes exist, no analysis was performed.

Exposure	1 Day	2 Days	3 Days	4 Days	5 Days	*F*	*p*
Dose (mg a.i./cm^2^)							
0.01	0.0 ± 0.0 C	0.0 ± 0.0 Cb	8.9 ± 2.0 B	25.6 ± 2.9 Ab	45.6 ± 3.8 Ac	96.4	<0.01
0.05	0.0 ± 0.0 D	0.0 ± 0.0 Db	15.6 ± 1.8 C	30.0 ± 5.3 Bb	62.2 ± 2.8 Ab	333.6	<0.01
0.1	0.0 ± 0.0 C	0.0 ± 0.0 Cb	18.9 ± 4.8 B	36.7 ± 2.9 Aab	71.1 ± 3.1 Aab	82.8	<0.01
0.5	0.0 ± 0.0 D	3.3 ± 1.7 Ca	23.3 ± 2.4 B	45.6 ± 2.4 ABa	80.0 ± 1.7 Aa	106.0	<0.01
*F*	-	4.0	2.6	4.8	21.3		
*p*	-	0.02	0.07	0.01	<0.01		

**Table 5:** Mean immediate mortality (% ± SE) of *Rhyzopertha dominica* adults exposed on concrete treated with chlorantraniliprole at four doses (0.01, 0.05, 0.1, and 0.5 mg a.i./cm^2^), for 1, 2, 3, 4, and 5 days. Within each row, means followed by the same uppercase letter are not significantly different (in all cases DF = 4, 44, Tukey–Kramer HSD test at *p* = 0.05). Within each column, means that are followed by the same lower-case letter are not significantly different (in all cases DF = 3, 35, Tukey–Kramer HSD test at *p* = 0.05). Where no letters exist, no significant differences were recorded.

Exposure	1 Day	2 Days	3 Days	4 Days	5 Days	*F*	*p*
Dose (mg a.i./cm^2^)							
0.01	0.0 ± 0.0 C	2.2 ± 1.5 BC	7.8 ± 2.8 B	18.9 ± 2.6 A	34.4 ± 3.8 Ab	29.8	<0.01
0.05	1.1 ± 1.1 C	4.4 ± 2.4 BC	8.9 ± 2.6 B	25.6 ± 2.9 A	40.0 ± 4.1 Aab	22.0	<0.01
0.1	2.2 ± 1.5 C	8.9 ± 2.6 B	15.6 ±2.4 AB	26.7 ± 3.3 A	43.3 ± 2.4 Aab	22.9	<0.01
0.5	4.4 ± 2.4 D	10.0 ± 3.3 CD	16.7 ± 4.1 BC	32.2 ± 4.3 AB	50.0 ± 4.1 Aa	13.2	<0.01
*F*	1.4	2.2	2.6	2.2	3.1		
*p*	0.25	0.11	0.07	0.11	0.04		

**Table 6:** Mean immediate mortality (% ± SE) of *Acarus siro* adults exposed on concrete treated with chlorantraniliprole at four doses (0.01, 0.05, 0.1, and 0.5 mg a.i./cm^2^), for 1, 2, 3, 4, and 5 days. Within each row, means followed by the same uppercase letter are not significantly different (in all cases DF = 4, 44, Tukey–Kramer HSD test at *p* = 0.05). Within each column, means that are followed by the same lower-case letter are not significantly different (in all cases DF = 3, 35, Tukey–Kramer HSD test at *p* = 0.05). Where no letters exist, no significant differences were recorded.

Exposure	1 Day	2 Days	3 Days	4 Days	5 Days	*F*	*p*
Dose (mg a.i./cm^2^)							
0.01	0.0 ± 0.0 Dc	7.8 ± 2.2 Cb	24.4 ± 3.4 Bb	38.9 ± 3.5 ABc	65.6 ± 2.9 Ac	67.7	<0.01
0.05	5.5 ± 1.8 Cb	17.8 ± 3.6 Bab	33.3 ± 5.0 ABab	50.0 ± 6.2 Abc	74.4 ± 3.4 Abc	20.2	<0.01
0.1	10.0 ± 1.7 Cab	27.8 ± 4.3 BCa	38.9 ± 4.6 ABab	62.2 ± 4.7 Aab	82.2 ± 2.8 Aab	14.5	<0.01
0.5	17.8 ± 3.2 Ca	35.6 ± 6.7 BCa	54.4 ± 7.5 ABa	72.2 ± 5.5 Aa	92.2 ± 2.2 Aa	13.4	<0.01
*F*	14.2	4.5	4.3	9.0	14.2		
*p*	<0.01	0.01	0.01	0.01	<0.01		

**Table 7:** Mean immediate mortality (% ± SE) of *Acarus siro* nymphs exposed on concrete treated with chlorantraniliprole at four doses (0.01, 0.05, 0.1, and 0.5 mg a.i./cm^2^), for 1, 2, 3, 4, and 5 days. Within each row, means followed by the same uppercase letter are not significantly different (in all cases DF = 4, 44, Tukey–Kramer HSD test at *p* = 0.05). Within each column, means that are followed by the same lower-case letter are not significantly different (in all cases DF = 3, 35, Tukey–Kramer HSD test at *p* = 0.05). Where no letters exist, no significant differences were recorded.

Exposure	1 Day	2 Days	3 Days	4 Days	5 Days	*F*	*p*
Dose (mg a.i./cm^2^)							
0.01	2.2 ± 1.5 Cb	17.8 ± 2.2 Bc	27.8 ± 3.2 ABb	37.8 ± 2.2 Ac	45.6 ± 3.4 Ac	53.2	<0.01
0.05	7.8 ± 1.5 Ca	24.4 ± 3.4 Bbc	38.9 ± 3.9 ABab	47.8 ± 3.6 ABbc	56.7 ± 3.3 Abc	22.2	<0.01
0.1	13.3 ± 1.7 Da	31.1 ± 3.1 Cab	40.0 ± 3.3 BCa	54.4 ± 3.4 ABab	64.4 ± 4.4 Aab	51.5	<0.01
0.5	16.7 ± 1.7 Da	36.7 ± 2.9 Ca	46.7 ± 1.7 BCa	61.1 ± 2.6 ABa	73.3 ± 3.7 Aa	73.2	<0.01
*F*	15.8	7.6	5.6	10.9	9.8		
*p*	<0.01	0.01	0.01	<0.01	0.01		

**Table 8:** ANOVA parameters for main effects and the associated interaction for delayed mortality of *Tribolium castaneum* adults and larvae, *Sitophilus oryzae* adults, *Rhyzopertha dominica* adults, and *Acarus siro* adults and nymphs (total DF = 202).

Effect	DF	Species	
Source		*F*	*p*
Species	5	1.5	0.21
Dose	3	8.1	<0.01
Species × dose	15	0.4	1.00

**Table 9:** Mean delayed mortality (% ± SE) of *Tribolium castaneum* adults and larvae, *Sitophilus oryzae* adults, *Rhyzopertha dominica* adults, and *Acarus siro* adults and nymphs exposed on concrete treated with chlorantraniliprole at four doses (0.01, 0.05, 0.1, and 0.5 mg a.i./cm^2^). Within each row, means followed by the same lowercase letter are not significantly different (in all cases DF = 3, 35, Tukey–Kramer HSD test at *p* = 0.05). Where no letters exist, no significant differences were recorded.

Dose (mg a.i./cm^2^)	0.01	0.05	0.1	0.5	DF	*F*	*p*
Species/Life Stage							
*T. castaneum* adults	91.0 ± 2.9	95.4 ± 3.1	97.8 ± 2.2	100.0 ± 0.0	3, 35	2.5	0.08
*T. castaneum* larvae	88.0 ± 4.2	93.5 ± 4.3	97.2 ± 2.8	100.0 ± 0.0	3, 25	1.2	0.32
*S. oryzae* adults	97.8 ± 2.2	98.2 ± 1.9	100.0 ± 0.0	100.0 ± 0.0	3, 35	0.7	0.58
*R. dominica* adults	86.6 ± 3.4 b	92.6 ± 2.6 ab	96.7 ± 2.4 a	98.6 ± 1.4 a	3, 35	4.3	0.01
*A. siro* adults	88.2 ± 6.0	92.6 ± 4.9	100.0 ± 0.0	100.0 ± 0.0	3, 32	1.9	0.15
*A. siro* nymphs	90.3 ± 4.2	92.6 ± 3.8	94.4 ± 3.7	96.3 ± 3.7	3, 35	0.4	0.75

## Data Availability

Data are contained within the article.
